# Results of Lung Transplantation for Cystic Fibrosis With Selected Donors Over 65 Years Old

**DOI:** 10.3389/ti.2023.11180

**Published:** 2023-06-12

**Authors:** Matthieu Glorion, Matthieu Sarsam, Antoine Roux, Marc Stern, Natalia Belousova, Julien Fessler, Ciprian Pricopi, Julien De Wolf, Clement Picard, Olivier Brugière, Sandra De Miranda, Dominique Grenet, Guillaume Tachon, Charles Cerf, Francois Parquin, Morgan Le Guen, Alain Chapelier, Alexandre Vallée, Edouard Sage

**Affiliations:** ^1^Service de Chirurgie Thoracique et Transplantation Pulmonaire, Hôpital Foch, Suresnes, France; ^2^ Service de Pneumologie, Hopital Foch, Suresnes, France; ^3^ Université Paris-Saclay, UVSQ, INRAE, VIM, Jouy-en-Josas, France; ^4^ Département d’Anesthésiologie, Hôpital Foch, Suresnes, France; ^5^Service de Réanimation, Hôpital Foch, Suresnes, France; ^6^ Unité Biométrie, Hôpital Foch, Suresnes, France

**Keywords:** lung transplantation, elderly donors, cystic-fibrosis, lung procurment, lung aging

## Abstract

Lung transplantation is limited by the shortage of suitable donors. Many programs have begun to use extended criteria donors. Donors over 65 years old are rarely reported, especially for young cystic fibrosis recipients. This monocentric study was conducted for cystic fibrosis recipients from January 2005 to December 2019, comparing two cohorts according to lung donor age (<65 years or ≥65 years). The primary objective was to assess the survival rate at 3 years using a Cox multivariable model. Of the 356 lung recipients, 326 had donors under 65 years, and 30 had donors over 65 years. Donors’ characteristics did not differ significantly in terms of sex, time on mechanical ventilation before retrieval, and partial pressure of arterial oxygen/fraction of inspired oxygen ratio. There were no significant differences in post-operative mechanical ventilation duration and incidence of grade 3 primary graft dysfunction between the two groups. At 1, 3, and 5 years, the percentage of predicted forced expiratory volume in 1 s (*p* = 0.767) and survival rate did not differ between groups (*p* = 0.924). The use of lungs from donors over 65 years for cystic fibrosis recipients allows extension of the donor pool without compromising results. Longer follow-up is needed to assess the long-term effects of this practice.

## Graphical Abstract

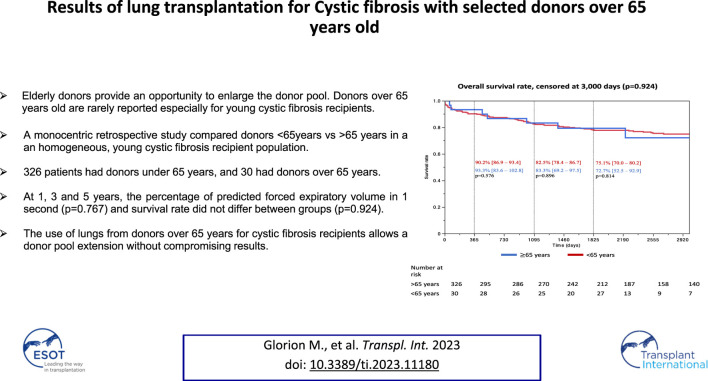

## Introduction

The number of lung transplants has increased steadily since the first transplant in 1986. While optimal donor criteria were [1] have been defined, the need to broaden them has gradually emerged with marginal donors [2–4]. Over time, donation methods have evolved, including donation after circulatory death and *ex vivo* lung perfusion (EVLP) strategies. This makes it possible to requalify certain grafts. However, the consequences of certain intrinsic selection criteria, such as age, remain uncertain.

Intuitively, the use of “young” grafts should be preferred for all recipients. However, data on lung aging are scarce and their consequences in transplantation are little known. Over the years, the donor age barrier has been gradually pushed, and the use of older grafts became a necessity.

At the beginning of the modern era of LT, it was considered that the ideal donor’s age should be <55 years [1]. This was based on retrospective analysis on the UNOS registry that showed a negative association between donor age and extended graft ischemic time, particularly in donors aged >55 years where ischemic time usually exceeded 6 h [5–7]. Howeverover time, LT indications were widened progressively, and optimal donors no longer corresponded to the emerging needs. For this reason, the boundaries of donor age, as well as other criteria, have been progressively modified.

The broadening of the age limit appears logical for diseases affecting older groups of recipients such as Idiopathic pulmonary fibrosis (IPF) and emphysema. However, for young recipients the question is crucial, as one of the pitfalls of allocation priority rules is that optimal transplants go to most urgent cases; often elderly patients, when some stable young patients are possibly offered grafts with expanded criteria.

It is essential to evaluate this practice in order to know the outcomes after LT with older donors. In our monocentric experience, we wanted to evaluate the effect of graft age in cystic fibrosis by comparing donors >65 and <65. We studied both survival and functional evolution.

## Patients and Methods

To assess the effect of donor age on outcomes after lung transplantation, a retrospective analysis of all bilateral lung transplants performed for cystic fibrosis was conducted in our center between January 2005 and December 2019.

Re-transplants were excluded. Two cohorts were defined according to donor age, one group with donors aged <65 years and the other group with donors aged ≥65.

Primary objective was the comparison of survival rate at 3 years between the two groups. Additionally, we conducted a secondary analysis, shown in a Supplementary Material, for survival rate at 5 years (Supplementary File S1). Secondary endpoints included the occurrence of grade III Primary Graft Dysfunction (PGD3) [8] at 24, 48, and 72 h following LT, the initial duration of mechanical ventilation (MV), the initial length of stay in the intensive care unit (ICU LOS), overall hospital length of stay (hospital LOS), the occurrence of graft neoplasm and Chronic lung allograft dysfunction (CLAD) onset at 3 and 5 years.

### Donors’ Lungs Allocation, Assessment and Procurement

All lungs were offered to our center by the *Agence de biomédecine* (ABM). Once the offer was accepted, the final assessment and retrieval were conducted by our procurement team. Assessment routinely included bronchoscopy, and macroscopic evaluation of the lung. Emphysematous lungs with bullae or rarefied parenchyma were rejected. *Ex vivo* lung perfusion with the Toronto technique [9] was used to evaluate and optimize marginal lungs. We retrospectively used the donor score [10] to assess the quality of the graft (range, 0–18; based on age, history of smoking, P/F Ratio, chest radiographs, and bronchoscopic findings).

Demographic data of donors and recipients were retrospectively recorded. Post-transplant follow-up parameters included lung function parameters at 1st; 2nd, 3rd as well as 5th postoperative year, such as forced expiratory volume in 1 s (FEV1) and ratio of FEV1/forced vital capacity (FVC). Predicted FEV1 was calculated for each recipient using the formula FEV1 = race × [(0.0395 × height) - (0.025 × age) - 2.6]. Since all recipients in the analyzed cohorts were Caucasian, “race” was substituted by “1” in the formula. The measured FEV1 was then expressed as the percentage predicted FEV1, and as a ratio to best post-operative FEV1 in order to assess intra-patient function evolution according last ISHLT consensus on CLAD [11]. Predicted total lung capacity (TLC) was calculated for each donor and recipient using the formula TLC = (height × 7.992)—7.081 for men and TLC = (height × 6.602)—5.791 for women.

Our surgical protocol for lung transplantation in CF consists of a sequential double lung transplant through a double anterolateral thoracotomy sparing the sternum [12]. Peripheral Veno-Arterial Extra Corporeal Membrane Oxygenation (ECMO) was initiated through femoral cannulation when intraoperative support was required. Post-operative ECMO was only used if PaO_2_/FiO_2_ < 100 mmHg or hemodynamic impairment [13].

Bronchial complications are described when major interventional treatment was necessary.

From an immunological point of view, regarding cellular rejection, we evaluated the A-score. A-score is calculated at specific time-points by adding the A-grades (perivascular mononuclear cell infiltrate graded A0–A4) of all transbronchial biopsies (TBB) performed up to the time-point, and dividing by the number of TBBs. Biopsies which were unable to be evaluated and given a grade of “Ax” are excluded from the calculation [14].

### Statistical Analysis

Continuous variables are presented as median and (25th–75th percentile), and were compared using a Mann Whitney non-parametric test. Categorical variables are presented as n (%) and were compared using a Chi-squared test or Fisher’s exact test.

Time to death (graft survival) and CLAD onset (freedom from CLAD) were estimated using the Kaplan-Meier method and compared by log-rank test.

Cox univariable regression was used to evaluate the association between clinical or biological factors, and 3-year survival for primary objective and at 5-year survival for secondary objective. The same analyses were performed for CLAD onset. Cox multivariable models were used to assess the association between the age group (≥65 or <65 years) and survival onset or CLAD onset with adjustment for potential confounding factors. Confounding factors with a significance of *p* < 0.05 on univariable analysis were selected for multivariable analyses.

We used an adjusted-repeated-measures mixed-model testing group outcome (donor age groups) for FEV1 and FEV1/FVC ratio changes over time.

Propensity score matching was performed with ratio 2:1 for control group as sensitivity analysis (Supplementary File S2).

For all analyses, *p* < 0.05 was considered statistically significant. Statistical analyses were performed using SAS software (version 9.4; SAS Institute, Carry, NC).

## Results

**FIGURE 1:**
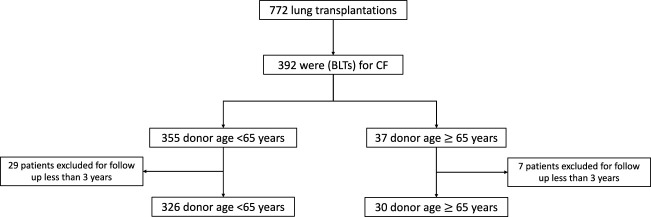
Flow chart.

Between January 2005 and December 2019, 772 lung transplants were performed at our center. Among them, 392 were (BLTs) for CF. We classified this population by donor age forming two groups. A total of 355 BLTs were performed with donors aged <65 years, and 37 with donors aged ≥65 years. Thirty-six patients were excluded due to a follow-up time under 3 years. Therefore, the analysis included 356 patients (326 with a donor aged <65 years, and 30 with a donor aged ≥65 years) (Figure 1).

Characteristics of donors and recipients are reported in Table 1. Donors had a median age of 45 (34–54) in <65 years group, and 68 (66–70) in ≥65 years group, *p* < 0.001. The elderly group included fewer smokers (*p* < 0.001), and higher Oto score [10] (*p* = 0.001) despite taking age into account. There was no difference in sex, P/F ratio, tracheal aspiration quality and MV duration before retrieval of the donor lungs.

**TABLE 1:** Characteristics of the study population.

Variables	<65 years (N = 326)	>65 years (N = 30)	*p*-Value
Donor age in years	45 (34–54)	68 (66–70)	<0.001
Donor sex (female)	130 (39.9)	16 (53.5)	0.155
Mechanical ventilation duration in days	2 (1–3)	1 (1–2)	0.551
Pao_2_/Fio_2_ at offer	375 (324–455)	385 (325–448)	0.817
Smoking history	137 (42.0)	3 (10.0)	<0.001
Tracheal aspiration quality			0.482
Clean	173 (54.8)	18 (63.7)	
Dirty	124 (39.2)	8 (29.6)	
Bloody	19 (6.0)	1 (3.7)	
Oto score	7 (4–9)	8 (7–10)	0.001
Recipient age in years	28.1 (23.9–33.8)	30.9 (25.7–40.9)	0.026
Recipient sex female	176 (54.0)	20 (66.7)	0.177
HELT	51 (15.6)	6 (20.0)	0.544
Time on waiting list	21 (7–58)	23 (7–57)	0.779
TLC ratio	1.07 (0.89–1.32)	1.09 (0.95–1.26)	0.798
Lobar transplant	27 (8.3)	0 (0.0)	0.026
CMV mismatch d+/r−	89 (27.3)	4 (1.3)	0.096
EVLP	35 (10.7)	2 (6.7)	0.459
Intraoperative ECMO	135 (41.4)	7 (23.3)	0.046
Post-operative ECMO	77 (23.6)	6 (20.0)	0.648
OT extubation	117 (35.9)	11 (36.7)	0.932
Tracheostomy	36 (18.4)	7 (24.1)	0.316
Duration of mechanical ventilation	2 (0–6)	1 (0–14)	0.836
Intensive care stay in days	6 (4–11)	9 (4.5–16.5)	0.161
Total hospital stay in days	28 (22–40)	30 (23.5–43)	0.736
PGD 3 at hours			
H24	81 (25.1)	6 (20.0)	0.277
H48	82 (25.4)	6 (20.0)	0.261
H72	58 (18.0)	5 (16.7)	0.546
Bronchial complications	78 (27.1)	1 (4.8)	0.009
Total ischemia time in minutes (N = 318)	368 (315–426)	400 (362–470)	0.029
Graft neoplasm	4 (1.23)	1 (3.33)	0.348
A score 1 year	0.111 (0–0.286)	0 (0–0.200)	0.095
A score 3 years	0.111 (0–0.250)	0 (0–0.208)	0.150
A score 5 years	0.105 (0–0.250)	0 (0–0.222)	0.149

Continuous data are presented as median (25th–75th percentile) and dichotomous data as n and percentage.

Recipients of donor lungs ≥65 years of age were significantly older than those receiving donor organs <65 years (*p* = 0.026). CMV mismatch tends to be more significant in ≥65 years than <65 years (*p* = 0.096). There was no difference observed in terms of waiting time on the list (*p* = 0.779), TLC ratio (*p* = 0.798), use of EVLP (*p* = 0.459) or need for high emergency lung transplant allocation (*p* = 0.544).

The elderly group showed higher total ischemia time in minutes than the younger group (*p* = 0.029). There was no difference between immediate post-operative extubation rate, primary graft dysfunction, length of MV duration, ICU or hospital stay. Interestingly, there were more bronchial complications in the younger donor group (*p* = 0.009).

**FIGURE 2:**
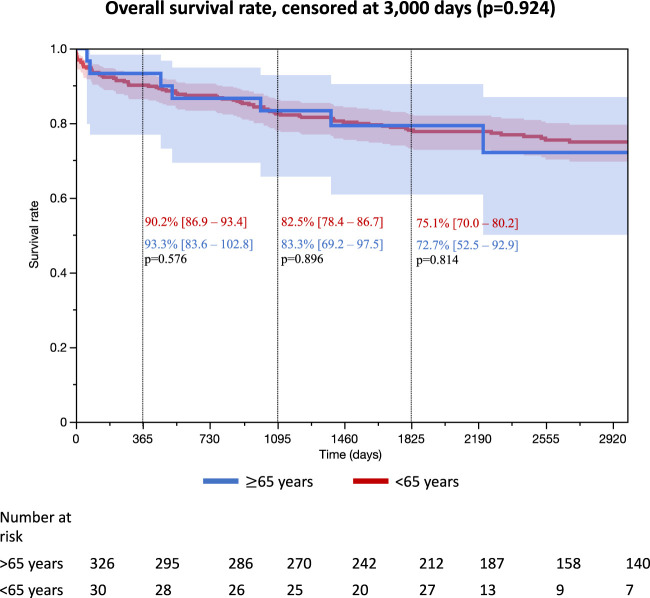
Kaplan Meier survival estimates *each proportion of survival rate was reported on the number of patients with follow up at the time of 1, 3, and 5 years.

Survival rates according to donor age are reported in Figure 2. No significant difference was observed between the two groups during the follow up censored at 3,000 days (*p* = 0.924), at 1 year (<65 years group: 90.2%, 95% CI [86.9–93.4] vs. 93.3% [83.6–102.8], *p* = 0.576), 3 years (<65 years group: 82.5% [78.4–86.7] vs. 83.3% [69.2–97.5], *p* = 0.896) and 5 years (N = 303, <65 years group: 75.1% [70.0–80.2] vs. 72.7% [52.5–92.9], *p* = 0.814).

In univariable analysis, donor age was not associated with survival rate at 3 years (HR = 0.94 [0.38–2.35], *p* = 0.896) and remained nonsignificant after adjustment for confounding factors (adjusted HR = 0.82 [0.13–5.11], *p* = 0.836) (Table 2). The same results were observed for survival rate at 5 years (N = 303, univariable HR = 1.11 [0.48–2.54], *p* = 0.814, and adjusted HR = 1.43 [0.48–4.25], *p* = 0.517) (Supplementary File S1).

**TABLE 2:** Cox univariable and multivariable analyses for survival rate at 3 years.

Variables	Univariable HR (95% CI)	*p*-Value	Adjusted HR (95% CI)	*p*-Value
Donor age ≥65 years	0.94 [0.38–2.35]	0.896	1.14 [0.13–10.19]	0.908
Donor sex (female)	0.99 [0.60–1.65]	0.989		
Mechanical ventilation duration in days	1.00 [0.93–1.05]	0.969		
Pao_2_/Fio_2_ at offer	1.00 [0.99–1.01]	0.772		
Smoking history	1.20 [0.73–1.99]	0.472		
Tracheal aspiration quality		0.205		
Clean	Ref.			
Dirty	0.88 [0.52–1.48]	0.636		
Bloody	0.24 [0.03–1.78]	0.164		
Oto score	0.96 [0.88–1.05]	0.375		
Recipient age in years	**0.96 [0.92–0.99]**	**0.009**	0.81 [0.69–0.95]	0.017
Recipient sex female	1.15 [0.69–1.90]	0.589		
HELT	**2.02 [1.14–3.56]**	**0.016**	9.75 [1.52–22.15]	0.014
Time on waiting list	1.00 [0.99–1.01]	0.649		
TLC ratio	**2.08 [1.01–3.88]**	**0.049**	0.69 [0.02–18.50]	0.866
Lobar transplant	**2.92 [1.48–5.76]**	**0.002**	1.04 [0.42–4.83]	0.976
CMV mismatch d+/r−	1.14 [0.66–1.97]	0.647		
EVLP	0.57 [0.21–1.56]	0.272		
Intraoperative ECMO	1.56 [0.95–2.56]	0.082		
Post-operative ECMO	**1.76 [1.03–2.99]**	**0.038**	0.02 [0.01–0.61]	0.023
OT extubation	0.82 [0.48–1.40]	0.476		
Tracheostomy	1.80 [0.88–3.71]	0.107		
Duration of mechanical ventilation	**1.01 [1.00–1.02]**	**0.028**	1.01 [1.00–1.02]	0.018
Intensive care stay in days	**1.03 [1.01–1.05]**	**<0.001**	1.07 [0.99–1.17]	0.059
Total hospital stay in days	1.00 [0.99–1.01]	0.051		
PGD 3 at hours				
H24	2.00 [1.19–3.39]	0.010		
H48	1.97 [1.16–3.32]	0.011		
H72	**3.30 [1.95–5.58]**	**<0.001**	17.15 [1.61–35.54]	0.019
Bronchial complications	**2.02 [1.15–3.55]**	**0.014**	5.47 [1.40–21.43]	0.011
A score 1 year	2.76 [0.70–9.58]	0.142		
A score 3 years	**6.17 [1.53–21.96]**	**0.007**	11.11 [0.48–25.74]	0.133
A score 5 years	6.88 [1.66–25.33]	0.005		
Graft neoplasm	—	0.999		
Total ischemia time in minutes	1.00 [0.99–1.01]	0.159		

Bold values represents p<0.05.

**FIGURE 3:**
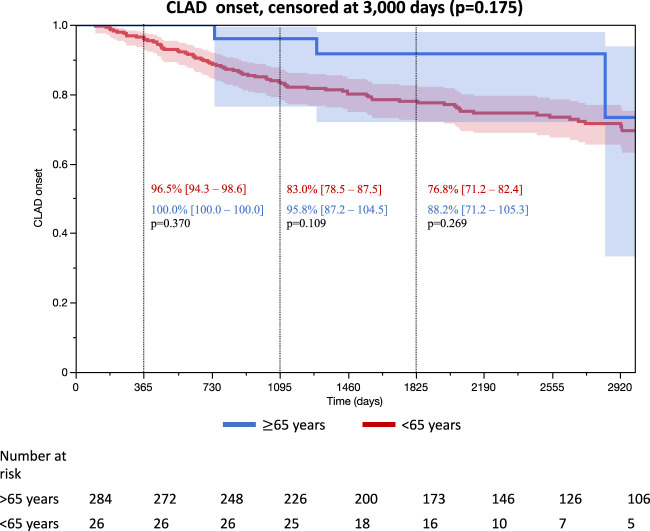
Occurrence of CLAD *each proportion of CLAD onset was reported on the number of patients with follow up at the time of 1, 3, and 5 years.

CLAD occurrence is reported in Figure 3 and did not differ with donor age during the follow up censored at 3,000 days (*p* = 0.175). In univariable analysis, donor age was not associated with CLAD occurrence at 3 years (for group ≥65 years, HR = 0.23 [0.03–1.65], *p* = 0.143) and remained nonsignificant after adjustment for confounding factors (for group ≥65 years, adjusted HR = 0.89 [0.11–7.03], *p* = 0.913) (Table 3). The same results were observed for CLAD occurrence at 5 years (N = 303, univariable HR = 0.46 [0.11–1.90], *p* = 0.284, and adjusted HR = 1.27 [0.28–5.82], *p* = 0.763) (Supplementary File S1).

**TABLE 3:** Cox univariable and multivariate analyses for CLAD onset at 3 years.

Variables	Univariable HR (95% CI)	*p*-Value	Adjusted HR (95% CI)	*p*-Value
Donor age ≥65 years	0.23 [0.03–1.65]	0.143	0.89 [0.11–7.03]	0.913
Donor sex (female)	0.96 [0.54–1.72]	0.891		
Mechanical ventilation duration in days	1.02 [0.96–1.07]	0.424		
Pao_2_/Fio_2_ at offer	1.00 [0.99–1.01]	0.538		
Smoking history	**1.94 [1.09–3.44]**	**0.024**	1.57 [0.63–3.96]	0.329
Tracheal aspiration quality		0.778		
Clean	Ref.			
Dirty	0.88 [0.47–1.68]	0.711		
Bloody	1.31 [0.47–3.77]	0.614		
Oto score	0.95 [0.86–1.05]	0.319		
Recipient age in years	**0.89 [0.84–0.94]**	**<0.001**	0.86 [0.69–0.93]	<0.001
Recipient sex female	0.62 [0.35–1.10]	0.103		
HELT	0.68 [0.27–1.71]	0.411		
Time on waiting list	1.00 [0.99–1.01]	0.572		
TLC ratio	0.91 [0.30–1.09]	0.859		
Lobar transplant	0.72 [0.17–2.97]	0.649		
CMV mismatch d+/r−	**2.31 [1.29–4.12]**	**0.005**	1.93 [0.78–4.77]	0.153
EVLP	0.66 [0.21–2.13]	0.487		
Intraoperative ECMO	1.22 [0.68–2.19]	0.491		
Post-operative ECMO	1.27 [0.59–2.72]	0.536		
OT extubation	1.02 [0.56–1.84]	0.935		
Tracheostomy	0.40 [0.10–1.79]	0.213		
Duration of mechanical ventilation	1.00 [0.99–1.01]	0.379		
Intensive care stay in days	1.00 [0.99–1.01]	0.803		
Total hospital stay in days	**1.01 [1.00–1.02]**	**0.045**	1.01 [1.00–1.03]	0.045
PGD 3 at hours				
H24	1.07 [0.54–2.10]	0.841		
H48	1.06 [0.53–2.14]	0.866		
H72	1.08 [0.48–2.42]	0.843		
Bronchial complications	**2.14 [1.13–4.05]**	**0.020**	2.36 [0.96–5.81]	0.061
A score 1 year	**3.92 [0.97**–**14.09]**	**0.055**		
A score 3 years	**12.29 [3.02**–**24.87]**	**<0.001**	2.39 [0.25–16.97]	0.411
A score 5 years	**14.83 [3.45**–**37.85]**	**<0.001**		
Graft neoplasm	1.22 [0.17–8.85]	0.844		
Total ischemia time in minutes	1.00 [0.99–1.01]	0.509		

Bold values represents p<0.05.

**FIGURE 4:**
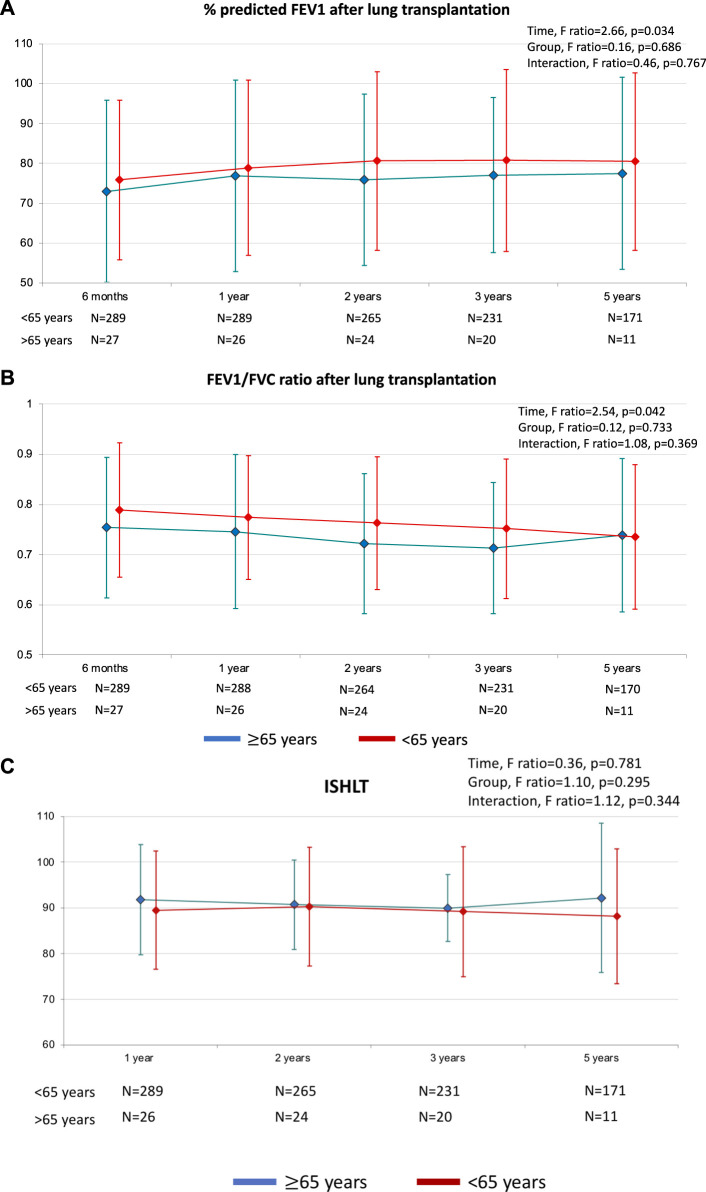
Post-operative spirometry results. **(A)** The percentage predicted forced expiratory volume in 1 s (FEV1), defined as measured FEV1 expressed as a percentage of the predicted FEV1. **(B)** FEV1/FVC: FEV1 measured/forced vital capacity. **(C)** ISHLT: FEV1 measured/best post operative FEV1.

The percentage of predicted FEV1 values were calculated to normalize the measured FEV1 and also expressed as a ratio to best post-operative FEV1 in order to assess intra-patient functional evolution. Mixed-model for repeated-measures of FEV1 during follow-up demonstrated no significant interaction between time and donor age group (*p* for interaction = 0.767 for predicted FEV1, *p* for interaction 0.344 for ratio to best post-operative FEV1). The same results were observed for obstructive impairment of lung function with FEV1/FVC (p for interaction = 0.369) (Figure 4).

### Sensitivity Analysis

When applying a propensity score match, allocating a matching ratio of 2:1 for the number of control patients, we found similar results. For survival rate at 3 years, univariable HR = 1.46 [0.46–4.61], *p* = 0.516, and after adjustment for covariates, adjusted HR = 0.91 [0.26–3.16], *p* = 0.880. For CLAD onset at 3 years, univariable HR = 0.70 [0.07–6.68], *p* = 0.746 and after adjustment for covariates an adjusted HR = 0.46 [0.10–2.18], *p* = 0.428 (Supplementary File S2).

## Discussion

In our single center retrospective study, a young cohort of 392 BLTs for CF was studied over a 15-year period. Grafts from donors aged 65 years or older accounted for 9.4% of the transplant volume, and resulted in no differences in outcomes compared to grafts from younger donors in our principal analysis at 3 years and in the secondary analysis at 5 years. These encouraging results generally reassure our practice and lead us to continue to accept lung graft offers from donors aged ≥65 years.

Additionally, in our study, we demonstrated no differences between the two groups in deterioration of lung function over time. Regarding susceptibility to cellular rejection, the older graft did not appear to modify the occurrence of events as estimated by A-score. Thus, there does not appear to be a difference in terms of the occurrence of chronic lung allograft dysfunction (CLAD). Another interesting point is that there is no difference in cancer occurrence in the graft, although the even rate is too low to provide sufficient statistical power in this analysis. This could be assessed in the future with a longer follow-up time.

### The Historical Point of View

Various experiences have been reported in the literature [15–17]. On one hand, in a 2007 retrospective study, De Perrot et al showed that the use of donors of >60 years of age was associated with lower 10-year survival [15]. These results were supported by Baldwin et al who reported their experience in 2015 [16]. On the other hand, in 2015, Sommer et al reported encouraging results with their retrospective study of donors aged >70 years in a cohort of COPD and restrictive patients. Interestingly, they found no survival difference but observed poorer lung function in restrictive recipients transplanted with older grafts [17]. Similarly, Hecker et al showed no survival differences with grafts over 65 years old [18].

Regarding these discrepancies, in a recent publication, Renard et al recommended caution with the use of elderly grafts, and preferential matching with elderly recipients [19].

Interestingly, one paper in the literature by Auråen et al [20]seems to have directly focused on the CF patient population but presents different conclusions, demonstrating a lower overall survival for donors over 55 years of age. These results were multicenteric from 5 Scandinavian centers, with a CF subgroup representing a sample size of 165 patients, which is smaller than that in our monocentric cohort.

### Cystic Fibrosis in France

The constitution of a donor/recipient pair calls for multiple compromises, the parameters of which are adjusted according to the severity of the recipient’s clinical condition. It goes without saying that in an emergency situation, such problems would not arise because the right graft is the one which is available to save the patient’s life.

In France the high emergency lung transplantation (HELT) system gives urgent patients priority access to optimal grafts [21]. With this pool being limited, patients on standard lists are therefore sometimes offered marginal grafts, and the choice comes down to a trade-off between the different parameters. It is in this context that our team used grafts> 65 years of age in this cohort of young patients.

Since January 2020, the problem has changed, as BLTs have become increasingly rare in this patient group thanks to the marketing of new CF therapeutics [22]**.**

### Physiological Data Regarding Aging Lungs

Although data on lung aging are limited, it is generally accepted that FEV1 decreases with age. This is due to changes in lung tissues, which result in larger alveoli without damage to their walls. This reduces alveolar surface tension and causes a decrease in the lungs’ elastic recoil, leading to a reduced maximum achievable flow during breathing.

Additionally, muscle performance and chest wall elasticity both decrease with age, resulting in an increased residual volume that counteracts any potential increase in total lung capacity (TLC) from reduced elastic recoil [23].

The consequences of the biological aspects of lung aging, such as telomere shortening, have yet to be fully understood.

### Tailored Graft Selection

The determinants of lung transplant survival are numerous. It is likely that the choice of donor is important from an immunological, viral (CMV), and size matching point of view. When possible, we tend to customize the choice of “the best” graft. However, taken alone, there is no certainty about the relevance of the age criterion when it comes to survival.

In our study, we found out that the older grafts were significantly better size-matched because no lobar transplant was performed in this group, there was less CMV mismatch, and preoperative plasmapheresis.

In this context of a tailored choice, it also seems interesting to consider the indication, as seen in the Sommer study, which demonstrated poorer functional results with elderly donors in the group of IPF patients in comparison with the COPD group. In our case, CF patients are examined, and they do not appear to have more functional impairment with elderly donors. Could the loss of elasticity of the lung tissue of older donors, advanced by Miller et al [23], be an explanation for the functional results of IPF patients.

### Limitations

This is a retrospective and monocentric study over an extended period during which transplant management practices have evolved. The use of older donors is more frequent in the most recent period and therefore this group has a shorter follow-up-period.

Furthermore, it is interesting to note that some poor prognostic factors at the time of organ selection, such as CMV mismatch or donor smoking history, although statistically non-significant, were more frequent in the group of younger donors. This suggests that there may be a likely allocation bias. This could be explained by a desire at the time of selection to avoid combining multiple risk factors.

A limitation of our study is that we do not present data on the presence of DSA and humoral rejection for which there were missing data for some of the cohort. Our management strategy has evolved over time and has been previously published [24].

From a statistical point of view, the groups are strongly imbalanced in terms of numbers.

### Strenghts of This Study

We present a homogenous cohort of young patients transplanted for CF. Despite decreasing numbers of LT in CF thanks to the development of new treatments, we keep updating our database rigorously and as a next step, a 10-year survival could be explored.

### Conclusion

Donor age alone should not be a reason to refuse a lung graft offer even in young recipients. While immediate and intermediate results do not show any significant statistical differences, long term results still need to be identified.

## Data Availability

The raw data supporting the conclusion of this article will be made available by the authors, without undue reservation.

## Abbreviations

BLT, bilateral lung transplantation; CF, cystic fibrosis; CLAD, chronic lung allograft dysfunction; ECMO, extracorporeal membrane oxygenation; EVLP, *ex vivo* lung perfusion; HELT, high emergency lung transplant; ICU, intensive care unit; IPF, idiopathic pulmonary fibrosis; LOS, length of stay; LT, lung transplantation; MV, mechanical ventilation; OS, overall survival; PGD, primary graft dysfunction, TLC, total lung capacity.
