# Supplementary material for: Results of Lung Transplantation for Cystic Fibrosis With Selected Donors Over 65 Years Old
Source: Transpl Int. 2023 Jun 12;36:11180. doi: 10.3389/ti.2023.11180 (PMC10316425; doi:10.3389/ti.2023.11180)
Supplement: Supplementary file 1 [file DataSheet1.docx]

Supplemental File 1 : Cox univariable and multivariable analyses for survival and CLAD onset at 5 years

Table 1: Characteristics of the population (N=303) and Cox univariable and multivariable analyses for survival at 5 years

| **variables** | **<65 years**  **(N=281)** | **>65 years (N=22)** | **P value** | **Univariable HR (95%CI)** | **P value** | **Adjusted HR (95% CI)** | **P value** |
| --- | --- | --- | --- | --- | --- | --- | --- |
| **Donor age ≥ 65 years** | - | - | - | **1.11 [0.48 – 2.54]** | **0.814** | 1.43 [0.48 – 4.25] | 0.517 |
| **Donor sex (female)** | 109 (38.8) | 10 (45.5) | 0.538 | 1.21 [0.77 – 1.91] | 0.398 |  |  |
| **Mechanical ventilation duration in days** | 2 [1 – 3] | 1 [1 – 2] | 0.972 | 1.02 [0.95 – 1.09] | 0.487 |  |  |
| **Pao2/Fio2 at offer** | 372 [320 – 456] | 392 [304 – 445] | 0.953 | 1.00 [0.99 – 1.01] | 0.442 |  |  |
| **Smoking history** | 115 (40.9) | 3 (13.6) | 0.012 | 1.16 [0.74 – 1.84] | 0.513 |  |  |
| **Tracheal aspiration quality** |  |  | 0.325 |  | 0.314 |  |  |
| **Clean** | 149 (54.6) | 15 (71.4) |  | Ref. |  |  |  |
| **Dirty** | 105 (38.5) | 5 (23.8) |  | 0.90 [0.55 – 1.45] | 0.653 |  |  |
| **Bloody** | 19 (6.9) | 1 (4.8) |  | 0.34 [0.08 – 1.39] | 0.133 |  |  |
| **Oto score** | 6 [4 – 9] | 9 [8 – 12] | 0.002 | 0.97 [0.91 – 1.05] | 0.507 |  |  |
| **Recipient age in years** | 28.4 [23.8 – 34] | 30.8 [26.5 – 40.7] | 0.147 | **0.97 [0.94 – 0.99]** | **0.044** | 0.96 [0.91 – 1.00] | 0.071 |
| **Recipient sex female** | 149 (53.0) | 14 (63.6) | 0.332 | 1.09 [0.69 – 1.71] | 0.716 |  |  |
| **HELT** | 48 (28.8) | 4 (18.2) | 0.895 | **1.81 [1.07 – 3.04]** | **0.025** | 2.06 [0.97 ) 4.34] | 0.058 |
| **Time on waiting list** | 22 [7 – 67] | 23 [9 – 88] | 0.988 | 0.99 [0.98 – 1.01] | 0.566 |  |  |
| **TLC ratio** | 1.09 [0.90 – 1.34] | 1.09 [0.90 – 1.24] | 0.715 | 1.64 [0.85 – 2.93] | 0.118 |  |  |
| **Lobar transplant** | 27 (9.6) | 0 (0) | 0.128 | **2.14 [1.13 – 4.07]** | **0.019** | 1.09 [0.30 – 3.97] | 0.901 |
| **CMV mismatch d+/r-** | 81 (28.8) | 2 (9.1) | 0.046 | 1.13 [0.69 – 1.85] | 0.620 |  |  |
| **EVLP** | 26 (9.3) | 1 (4.6) | 0.456 | 0.83 [0.36 – 1.92] | 0.670 |  |  |
| **Intraoperative ECMO** | 113 (40.2) | 5 (22.7) | 0.105 | 1.27 [0.81 – 1.99] | 0.305 |  |  |
| **Post-operative ECMO** | 66 (23.5) | 4 (18.2) | 0.569 | 1.55 [0.95 – 2.55] | 0.082 |  |  |
| **OT extubation** | 99 (35.2) | 6 (27.3) | 0.450 | 0.79 [0.48 – 1.29] | 0.356 |  |  |
| **Tracheostomy** | 33 (18.9) | 6 (28.6) | 0292 | 1.50 [0.78 – 2.89] | 0.225 |  |  |
| **Duration of mechanical ventilation** | 2 [0 – 7] | 2 [0 – 16.5] | 0.964 | **1.01[1.00 – 1.02]** | **0.017** | 1.01 [1.00 – 1.02] | 0.045 |
| **Intensive care stay in days** | 6 [4 – 11] | 7 [4.5 – 16.5] | 0.167 | **1.03 [1.02 – 1.05]** | **<0.001** | 1.01 [0.98 – 1.04] | 0.293 |
| **Total hospital stay in days** | 29 [22 – 44] | 30 [22.5 – 39.5] | 0.582 | 1.01 [1.00 – 1.02] | 0.041 |  |  |
| **PGD 3at 72 hours** |  |  |  |  |  |  |  |
| **H24** | 71 (25.5) | 4 (18.2) | 0.448 | 1.89 [1.18 – 3.06] | 0.009 |  |  |
| **H48** | 74 (26.5) | 4 (18.2) | 0.390 | 1.78 [1.10 – 2.87] | 0.018 |  |  |
| **H72** | 50 (17.9) | 3 (13.6) | 0.612 | **3.17 [1.96 – 5.14]** | **<0.001** | 3.07 [1.29 – 7.34] | 0.014 |
| **Bronchial complications** | 77 (28.3) | 1 (5.0) | 0.023 | **1.88 [1.14 – 3.10]** | **0.013** | 1.66 [0.80 – 3.46] | 0.175 |
| **Graft neoplasm** | 4 (1.42%) | 1 (4.55%) | 0.268 | **-** | **0.999** |  |  |
| **A score 1 year** | 0.125 [0-0.286] | 0 [0-0.225] | 0.232 | **1.41 [0.39 – 4.56]** | **0.585** |  |  |
| **A score 3 years** | 0.111 [0-0.250] | 0 (0-0.250] | 0.351 | **2.75 [0.73 – 9.28]** | **0.118** |  |  |
| **A score 5 years** | 0.111 [0-0.250] | 0 (0-0.236] | 0.358 | **2.83 [0.72 – 9.93]** | **0.117** |  |  |
| **Total ischemia** | 365 [312-430] | 420 [363-475] | 0.030 | **1.00 [0.99 – 1.01]** | **0.245** |  |  |

Table 2: Cox univariable and multivariable analyses for CLAD onset at 5 years (N=303)

| **Variables** | **Univariate HR (95%CI)** | **P value** | **Adjusted HR (95% CI)** | **P value** |  |
| --- | --- | --- | --- | --- | --- |
| **Donor age ≥ 65 years** | **0.46 [0.11 – 1.90]** | **0.284** | **1.27 [0.28 – 5.82]** | **0.763** |  |
| **Donor sex (female)** | 1.26 [0.74 – 2.15] | 0.399 |  |  |  |
| **Mechanical ventilation duration in days** | 0.99 [0.90 – 1.01] | 0.877 |  |  |  |
| **Pao2/Fio2 at offer** | 1.00 [0.99 – 1.01] | 0.302 |  |  |  |
| **Smoking history** | 1.54 [0.90 – 2.62] | 0.116 |  |  |  |
| **Tracheal aspiration quality** |  | 0.728 |  |  |  |
|  |  |  |  |  |  |
| **Clean** | Ref. |  |  |  |  |
| **Dirty** | 0.84 [0.46 – 1.53] | 0.571 |  |  |  |
| **Bloody** | 1.22 [0.47 – 3.15] | 0.679 |  |  |  |
| **Oto score** | 0.94 [0.86 – 1.02] | 0.153 |  |  |  |
| **Recipient age in years** | **0.91 [0.87 – 0.95]** | **<0.001** | 0.90 [0.84 – 0.96] | 0.003 |  |
| **Recipient sex female** | 0.66 [0.38 – 1.12] | 0.124 |  |  |  |
| **HELT** | 1.01 [0.48 – 2.15] | 0.968 |  |  |  |
| **Time on waiting list** | 1.00 [0.99 – 1.01] | 0.404 |  |  |  |
| **TLC ratio** | 1.07 [0.42 – 2.39] | 0.879 |  |  |  |
| **Lobar transplant** | 1.02 [0.37 – 2.83] | 0.966 |  |  |  |
| **CMV mismatch d+/r-** | **2.14 [1.29 – 4.12]** | **0.005** | 2.14 [0.91 – 5.07] | 0.083 |  |
| **EVLP** | 0.57 [0.19 – 1.82] | 0.345 |  |  |  |
| **Intraoperative ECMO** | 1.09 [0.63 – 1.88] | 0.766 |  |  |  |
| **Post-operative ECMO** | 0.64 [0.29 – 1.42] | 0.274 |  |  |  |
| **OT extubation** | 0.85 [0.48 – 1.50] | 0.576 |  |  |  |
| **Tracheostomy** | 0.71 [0.25 – 2.03] | 0.519 |  |  |  |
| **Duration of mechanical ventilation** | 1.00 [0.99 – 1.01] | 0.352 |  |  |  |
| **Intensive care stay in days** | 1.01 [0.98 – 1.05] | 0.373 |  |  |  |
| **Total hospital stay in days** | **1.02 [1.01 – 1.03]** | **0.016** | **1.02 [1.01 – 1.03]** | **0.022** |  |
| **PGD 3at 72 hours** |  |  |  |  |  |
| **H24** | 1.00 [0.52 – 1.93] | 0.995 |  |  |  |
| **H48** | 0.94 [0.48 – 1.81] | 0.834 |  |  |  |
| **H72** | 0.91 [0.39 – 2.12] | 0.826 |  |  |  |
| **Bronchial complications** | **2.02 [1.14 – 3.60]** | **0.016** | **2.56 [1.12 – 5.89]** | **0.027** |  |
| **A score 1 year** | **3.07 [0.83 – 10.27]** | **0.089** |  |  |  |
| **A score 3 years** | **7.86 [2.06 – 27.23]** | **0.003** |  |  |  |
| **A score 5 years** | **8.91 [2.20 – 33.06]** | **0.002** | **2.59 [0.59 – 9.88]** | **0.179** |  |
| **Graft neoplasm** | **1.77 [0.43 – 7.27]** | **0.438** |  |  |  |
| **Total ischemia** | **1.00 [0.99 – 1.01]** | **0.957** |  |  |  |

Supplementary file 2

**Propensity score sensitivity analysis**

**Methodology**

Propensity score to estimate the probability that patients would be selected for age over 65 years or not, was calculated using a multivariate logistic regression model to adjust for between-group differences in baseline characteristics based on covariates and factors. The inverse probability of treatment weighted (IPTW) methodology was performed to adjust for between-group differences, which were obtained from the propensity score [1]. Using the IPTW methodology approach, the weights for patients who were more than 65 years were set in proportion to the inverse of the propensity score; for the control group, the weights were set to the inverse of (1-propensity score).

Covariates included in the model were donor sex, recipient sex, age at the time of transplantation, donor smoking history, CMV mismatch, P/F ratio, ECMO, Oto score, date of operation, waiting time, HELT, EVLP, OR extubation

Then, we applied a matching ratio 2:1 for control group.

References

[1] Haukoos JS, Lewis RJ. The propensity score. JAMA. 2015;314(15):1637–8.

Table 1: Descriptive table of the propensity score population

| **variables** | **<65 years**  **(N=60)** | **>65 years**  **(N=30)** | **P value** |
| --- | --- | --- | --- |
| **Donor’s age in years** | 53 (44 - 60) | 68 (66 - 70) | <0.001 |
| **Donor sex (female)** | 31 (51.7) | 16 (53.5) | 0.881 |
| **Mechanical ventilation duration in days** | 2 (1-4) | 1 (1-2) | 0.519 |
| **Pao2/Fio2 at offer** | 371 (322 - 449) | 385 (325 - 448) | 0.833 |
| **Smoking history** | 7 (11.7) | 3 (10.0) | 0.813 |
| **Tracheal aspiration quality** |  |  | 0.093 |
| **Clean** | 24 (41.4) | 18 (63.7) |  |
| **Dirty** | 31 (43.4) | 8 (29.6) |  |
| **Bloody** | 3 (5.2) | 1 (3.7) |  |
| **Oto score** | 9 (7 - 11) | 8 (7 - 10) | 0.745 |
| **Recipient age in years** | 32.3 (25.6 – 38.5) | 30.9 (25.7 – 40.9) | 0.754 |
| **Recipient sex female** | 41 (68.3) | 20 (66.7) | 0.873 |
| **HELT** | 11 (18.33) | 6 (20.0) | 0.849 |
| **Time on waiting list** | 24 (5 - 67) | 23 (7 - 57) | 0.954 |
| **TLC ratio** | 1.04 (0.88 – 1.22) | 1.09 (0.95 – 1.26) | 0.427 |
| **Lobar transplant** | 2 (3.3) | 0 (0.0) | 0.311 |
| **CMV mismatch d+/r-** | 10 (16.7) | 4 (13.3) | 0.681 |
| **EVLP** | 5 (8.3) | 2 (6.7) | 0.781 |
| **Intraoperative ECMO** | 18 (30.0) | 7 (23.3) | 0.506 |
| **Post-operative ECMO** | 12 (20.0) | 6 (20.0) | 1.000 |
| **OT extubation** | 19 (31.7) | 11 (36.7) | 0.635 |
| **Tracheostomy** | 6 (10.2) | 7 (24.1) | 0.083 |
| **Duration of mechanical ventilation** | 1.5 (0 - 4) | 1 (0 - 14) | 0.421 |
| **Intensive care stay in days** | 6 (4 - 11) | 9 (4.5 – 16.5) | 0.837 |
| **Total hospital stay in days** | 30 (24 - 39) | 30 (23.5 - 43) | 0.914 |
| **PGD 3at 72 hours** |  |  |  |
| **H24** | 13 (21.7) | 6 (20.0) | 0.854 |
| **H48** | 15 (25.0) | 6 (20.0) | 0.597 |
| **H72** | 10 (16.7) | 5 (16.7) | 1.000 |
| **Bronchial complications** | 9 (20.0) | 1 (4.8) | 0.108 |
| **Total ischemia time in minutes** | 382 (340 – 434) | 400 (362 – 470) | 0.343 |
| **Graft neoplasm** | 0 (0.0%) | 1 (3.33%) | 0.155 |
| **A score 1 year** | 0 (0-0.229) | 0 (0-0.200) | 0.449 |
| **A score 3 years** | 0 (0-0.200) | 0 (0-0.208) | 0.714 |
| **A score 5 years** | 0 (0-0.200) | 0 (0-0.222) | 0.762 |
| **Graft neoplasm** | 0 (0%) | 1 (3.3%) | 0.155 |

Table 2: Date of surgery

|  | **Group<65 years** |  | **Group>65 years** | **P =0.975** |
| --- | --- | --- | --- | --- |
| **Date of surgery** | **Number** | **%** | **Number** | **%** |
| 2008 | 1 | 1.67% | 1 | 3.33% |
| 2011 | 5 | 8.33% | 1 | 3.33% |
| 2012 | 2 | 3.33% | 2 | 6.67% |
| 2013 | 7 | 11.67% | 3 | 10.00% |
| 2014 | 7 | 11.67% | 3 | 10.00% |
| 2015 | 8 | 13.33% | 3 | 10.00% |
| 2016 | 11 | 18.33% | 5 | 16.67% |
| 2017 | 8 | 13.33% | 5 | 16.67% |
| 2018 | 8 | 13.33% | 4 | 13.33% |
| 2019 | 3 | 5.00% | 3 | 10.00% |

Table 3: Analysis for survival rate at 3 years

| **Variables** | **Univariable HR (95%CI)** | **P value** | **Adjusted HR (95% CI)** | **P value** |
| --- | --- | --- | --- | --- |
| **Donor age ≥ 65 years** | 1.46 [0.46 – 4.61] | 0.516 | 0.91 [0.26 – 3.16] | 0.880 |
| **Donor sex (female)** | 0.79 [0.25 – 2.51] | 0.699 |  |  |
| **Mechanical ventilation duration in days** | 0.96 [0.73 – 1.12] | 0.706 |  |  |
| **Pao2/Fio2 at offer** | 0.99 [0.98 – 1.01] | 0.287 |  |  |
| **Smoking history** | 1.85 [0.41 – 8.44] | 0.428 |  |  |
| **Tracheal aspiration quality** |  | 0.157 |  |  |
|  |  |  |  |  |
| **Clean** | Ref. |  |  |  |
| **Dirty** | 0.26 [0.05 – 1.31] | 0.103 |  |  |
| **Bloody** | - | 0.999 |  |  |
| **Oto score** | 1.06 [0.85 – 1.32] | 0.623 |  |  |
| **Recipient age in years** | 0.96 [0.90 – 1.02] | 0.237 |  |  |
| **Recipient sex female** | 2.43 [0.53 – 11.12] | 0.250 |  |  |
| **HELT** | 5.21 [1.67 – 16.17] | 0.004 | 6.86 [1.64 – 28.74] | 0.008 |
| **Time on waiting list** | 0.98 [0.94 – 0.99] | 0.013 |  |  |
| **TLC ratio** | 2.65 [0.23 – 23.64] | 0.409 |  |  |
| **Lobar transplant** | 6.57 [0.84 – 21.22] | 0.157 |  |  |
| **CMV mismatch d+/r-** | 0.49 [0.06 – 3.78] | 0.493 |  |  |
| **EVLP** | - | 0.999 |  |  |
| **Intraoperative ECMO** | 2.92 [0.94 – 9.07] | 0.063 |  |  |
| **Post-operative ECMO** | 1.47 [0.40 – 5.45] | 0.560 |  |  |
| **OT extubation** | 0.37 [0.08 – 1.71] | 0.204 |  |  |
| **Tracheostomy** | 3.26 [0.98 – 10.84] | 0.076 |  |  |
| **Duration of mechanical ventilation** | 1.06 [1.03 – 1.09] | <0.001 | 1.06 [1.02 – 1.12] | 0.007 |
| **Intensive care stay in days** | 1.03 [1.02 – 1.06] | <0.001 | 1.01 [0.97 – 1.05] | 0.533 |
| **Total hospital stay in days** | 1.04 [1.01 – 1.06] | 0.003 |  |  |
| **PGD 3at 72 hours** |  |  |  |  |
| **H24** | 1.36 [0.37 – 5.05] | 0.640 |  |  |
| **H48** | 1.18 [0.32 – 4.37] | 0.802 |  |  |
| **H72** | 1.91 [0.52 – 7.04] | 0.332 |  |  |
| **Bronchial complications** | 1.95 [0.39 – 9.67] | 0.413 |  |  |
| **A score 1 year** | 0.43 [0.01 – 11.71] | 0.677 |  |  |
| **A score 3 years** | 0.28 [0.01 – 11.12] | 0.577 |  |  |
| **A score 5 years** | 0.28 [0.01 – 13.43] | 0.571 |  |  |
| **Graft neoplasm** | - | 0.999 |  |  |

Table 4: analysis for CLAD onset at 3 years

| **Variables** | **Univariable HR (95%CI)** | **P value** | **Adjusted HR (95% CI)** | **P value** |
| --- | --- | --- | --- | --- |
| **Donor age ≥ 65 years** | 0.70 [0.07 – 6.68] | 0.746 | 0.46 [0.10 2.18] | 0.428 |
| **Donor sex (female)** | 0.88 [0.12 – 6.27] | 0.901 |  |  |
| **Mechanical ventilation duration in days** | 0.78 [0.25 – 1.6] | 0.357 |  |  |
| **Pao2/Fio2 at offer** | 0.98 [0.96 – 1.01] | 0.061 |  |  |
| **Smoking history** | 1.85 [0.41 – 8.44] | 0.428 |  |  |
| **Tracheal aspiration quality** |  | 0.782 |  |  |
|  |  |  |  |  |
| **Clean** | Ref. |  |  |  |
| **Dirty** | 1.06 [0.15 – 7.56] | 0.949 |  |  |
| **Bloody** | - | 0.999 |  |  |
| **Oto score** | 1.24 [0.85 – 1.92] | 0.274 |  |  |
| **Recipient age in years** | 0.89 [0.76 – 1.01] | 0.113 |  |  |
| **Recipient sex female** | - | 0.999 |  |  |
| **HELT** | 1.82 [0.19 – 17.45] | 0.624 |  |  |
| **Time on waiting list** | 0.96 [0.88 – 1.01] | 0.084 |  |  |
| **TLC ratio** | 3.66 [0.37 – 6.65] | 0.121 |  |  |
| **Lobar transplant** | - | 1.000 |  |  |
| **CMV mismatch d+/r-** | - | 0.999 |  |  |
| **EVLP** | - | 0.999 |  |  |
| **Intraoperative ECMO** | - | 0.999 |  |  |
| **Post-operative ECMO** | - | 0.999 |  |  |
| **OT extubation** | 1.78 [0.25 – 12.70] | 0.564 |  |  |
| **Tracheostomy** | - | 0.999 |  |  |
| **Duration of mechanical ventilation** | 1.02 [0.88 – 1.10] | 0.749 |  |  |
| **Intensive care stay in days** | 1.00 [0.89 – 1.05] | 0.931 |  |  |
| **Total hospital stay in days** | 0.99 [0.87 – 1.05] | 0.727 |  |  |
| **PGD 3at 72 hours** |  |  |  |  |
| **H24** | - | 0.999 |  |  |
| **H48** | - | 0.999 |  |  |
| **H72** | - | 0.999 |  |  |
| **Bronchial complications** | 2.40 [0.25 – 23.11] | 0.483 |  |  |
| **A score 1 year** | 3.76 [0.01 – 15.70] | 0.578 |  |  |
| **A score 3 years** | 2.03 [0.01 – 15.08] | 0.794 |  |  |
| **A score 5 years** | 2.08 [0.01 – 17.65] | 0.801 |  |  |
| **Graft neoplasm** | - | 0.999 |  |  |
